# Who Shall Be Born

**Published:** 1913-06

**Authors:** 


### Who Shall Be Born.

The investigation of the family history of each of the 400 defectives sheltered in the Vineland Training School, Vineland, N. J., has revealed the lamentable state of ignorance which exists in the minds of even our more enlightened citizens in regard to the symptoms which are characteristic of feeble-mindedness. Society seems unaware that such degenerates should not be allowed to marry, or that where illegitimate unions have been formed the simple performance of the marriage rite before legally authorized functionaries does not in the least protect society from the venom of the race.

A striking instance of this lack of public recognition of defectives came to the notice of the institution in connection with a little girl of seven who was brought to us some fifteen years back.

On investigation it was learned that the child had been born in an almshouse. Her mother, pretty, attractive, had formed an attachment for a man in the neighborhood and the rumor was that they were engaged. Nothing came of it, however, for she was poor and was put to service with a family in a distant city. No one thought of her as feeble-minded; no one thought much about her at all, for her family had sunk so far as scarcely to emerge above the social level. The sad story of her mother, which I shall tell—the grandmother of the girl at Vineland—had been forgotten, and the busy world went on its way intent upon cares and interests of its own. But it was only a few months before she came back, bringing with her the burden of approaching motherhood. Her mother, crushed under her own load of misery, was dying or dead, and the daughter went to the almshouse. Let no one suppose that this was tragic for her. Suffering comes only with intelligence, a sense of shame only with the power to grasp an ideal, and to realize that we have fallen below it. In her case, both conditions were wanting. Like an irrational creature who had followed a blind impulse, and as blindly accepted her fate, understanding nothing, learning nothing from her fall, which in her case was no fall at all.

Previous to these happenings, the respectable community in which they took place had been roused to indignation on learning that her father had been holding criminal intercourse with one of his own daughters. He was a degenerate, and when he had been put in jail the public wrath was satisfied. No one thought of his wife, who, though she belonged to a good family, had lost all social recognition through her unfortunate marriage with this man of unknown ancestry. Feeble in health, weak in will, overworked, and, above all, broken-hearted, she had not proved the dominant factor in the union. All she could do was to transmit enough of her own gentle, refined nature to her defective children to make them a more dangerous social element than they could otherwise have been. So she sickened and died, and it was in an almshouse that the little grandchild was born with whom we must reckon in the generation now approaching maturity.

At this point the respectable community began to take an interest in the daughter, now a young mother. A humane though misguided feeling led one of its members to remove the mother and her baby from the almshouse, taking them into her own home. The step at first seemed admirable and was applauded on every side. The young woman was perfectly honest, strong, willing and trainable in household affairs. There was something about her large, brown, appealing eyes that went to one's heart, while her gentle and unobtrusive ways won the approval of her mistress and the interest of her friends. But this was only for a time. It was not long before a strange look came into her eyes; her manners changed; she was not steady at her post; even her little child ceased to hold her, and she would be off and away no one knew where. The mistress, now deeply interested in the welfare of her charge, sought by every means in her power to bring the young woman back to her former self, but in vain. Failing here, she next sought out the cause of the change and found it in the person of a low, degraded fellow, recognized in the community as half-witted as well as alcoholic, besides being subject to strange drunken fits. Still hoping to save the girl, she attempted further to interfere, but received only insults in return. Feeling, rightly enough, that something ought to be done, she decided upon what seemed to her the only alternative, that of forcing the young people to marry. Both were willing to do this, since some one cared to bother with the arrangements, which meant nothing to them. Lawyer and minister were promptly summoned and the pair duly recognized as man and wife before the law.

In a little cabin down the road, the already deserted wife brought her second baby into the world. Except for the constant care of her former mistress, mother and child must have perished, for the winter was hard and the husband did nothing towards their support.

But our good woman was not at the end of her resources; she had seen the couple married and she intended to see that the husband took care of his family. After infinite trouble and annoyance, she succeeded in getting the pair engaged to work on the land of an unmarried farmer living some distance back in the country and away from any settlement. There it was hoped they would learn to attend to their own affairs and grow into respectability. It was on this farm that a second child was born to the couple, so that the family now numbered three.

But with all our good woman's foresight, with all her honest intentions, she could not have hit upon a more ill-advised scheme. The farmer in question, though himself not of normal intelligence, was good-looking and far superior in every way to the drunken imbecile to whom the girl had been married. More than this, he proved to be the man to whom report had said she had earlier been engaged. So it was not long before another child was to come to the farm, which the husband and the farmer each referred to the other, and which both consequently refused to claim.

The situation had become for the third time tragic, and our good woman felt she must again intervene. She more than suspected the farmer's guilt, and was indignant at his attitude. Reflecting on the husband's character and finding that it had always been unfit, she determined to see the pair divorced, and the woman then married to the farmer, who would thus be obliged to undertake her support. The determination was put into execution, though not until the two children born of the first marriage had been placed in a home, the farmer stoutly refusing to provide for them. The mother, however, sought to keep her oldest child with her, though in this she was not successful. Very soon she brought the little illegitimate girl to the woman who had interfered so much in her life, explaining that there would be no peace in the home while the child remained.

It was a really wise move, the one which our good woman next made, that of applying to have the child received into the Vineland institution. It had begun to dawn upon her that the family was not normal, and that special training was needed. In this way the little seven-year-old found entrance to the sphere in which she rightly belonged.

But the mother's story was not yet at an end. Her union this time proved successful. She was satisfied with her husband and he apparently with her. Her defects in housekeeping and personal tidiness did not wear on his dulled intelligence, while he possessed capacity enough to run his farm and provide for his constantly increasing family. Today five strangely interesting, yet strikingly defective children grace his home. The oldest girl is on the point of being put out to service.

One is appalled at the thought! Will some clean youth be attracted to her (for she is attractive, and only a trained eye could readily detect her deficiency) and so bring disaster upon himself and his home? Or will she sink to the lowest level of her kind, and add to the horror of degradation and crime with which the land abounds? Heaven forbid. Yet one of these fates surely awaits her, while society stands passively by. No one can be brought face to face with a fact so apparent without feeling that something must be done and done at once, if this girl and thousands of similarly defective girls are to be saved from themselves, and society saved from the evils they unwittingly engender.

This is indeed but an isolated case, yet in this girl's family alone seven other deficient boys and girls are growing to manhood and womanhood, each having the same tendencies, bearing the same taint, while from the families of her mother's deficient brothers and sisters other children of like grade are advancing swiftly on the selfsame road.—*The Survey,* September 28, 1912.

